# Moderate Alcohol Intake Changes Visual Perception by Enhancing V1 Inhibitory Surround Interactions

**DOI:** 10.3389/fnins.2021.682229

**Published:** 2021-07-05

**Authors:** Huan Wang, Zhengchun Wang, Yifeng Zhou, Tzvetomir Tzvetanov

**Affiliations:** ^1^Hefei National Laboratory for Physical Sciences at Microscale, School of Life Sciences, University of Science and Technology of China, Hefei, China; ^2^The Affiliated People’s Hospital of Ningbo University, Ningbo, China; ^3^State Key Laboratory of Brain and Cognitive Science, Institute of Biophysics, Chinese Academy of Sciences, Beijing, China; ^4^Anhui Province Key Laboratory of Affective Computing and Advanced Intelligent Machine, School of Computer and Information, Hefei University of Technology, Hefei, China; ^5^NEUROPSYPHY Tzvetomir TZVETANOV EIRL, Horbourg-Wihr, France

**Keywords:** moderate alcohol, surround suppression, tilt illusion, inhibitory processing, V1

## Abstract

Moderate alcohol consumption is considered to enhance the cortical GABA-ergic inhibitory system and it also variously affects visual perception. However, little behavioral evidence indicates changes of visual perception due to V1 modulated by alcohol intoxication. In this study, we investigated this issue by using center-surround tilt illusion (TI) as a probe of V1 inhibitory interactions, by taking into account possible higher-order effects. Participants conducted TI measures under sober, moderate alcohol intoxication, and placebo states. We found alcohol significantly increased repulsive TI effect and weakened orientation discrimination performance, which is consistent with the increase of lateral inhibition between orientation sensitive V1 neurons caused by alcohol intoxication. We also observed no visible changes in the data for global orientation processing but a presence of global attentional modulation. Thus, our results provide psychophysics evidence that alcohol changed V1 processing, which affects visual perception of contextual stimuli.

## Introduction

The visual system is a selective and primary target of acute alcohol effects (Esposito et al., 2010; Abrahao et al., 2017). Previous research has indicated various changes in visual perception caused by alcohol consumption, such as spatial frequency discrimination (Watten et al., 1998), contrast sensitivity (Nicholson et al., 1995; Cavalcanti-Galdino et al., 2014; Timney et al., 2016), eye accommodation and vergence (Hill and Toffolon, 1990), visual acuity (Wilson and Mitchell, 1983; Hill and Toffolon, 1990), or motion processing (MacArthur and Sekuler, 1982; Gummel et al., 2012). Primary visual cortex (V1) is the earliest cortical processing stage in the hierarchical organization of the visual system, and its circuit connectivity and neuronal response properties are well understood (Bijanzadeh et al., 2018). Neurophysiological research has reported the negative effects of acute alcohol exposure on the response properties of visual area 17 of cat (Chen et al., 2010), functional magnetic resonance imaging (fMRI) revealed a strong enhancement of spontaneous BOLD fluctuations in V1 in an acute alcoholic state (Esposito et al., 2010), and magnetoencephalography recordings (Campbell et al., 2014) showed that stimulus-induced Gamma oscillations in human V1 were also strongly modulated by alcohol consumption.

Surround suppression (SS) is a canonical cortical computation (Bijanzadeh et al., 2018), where stimuli beyond the classical receptive field (RF) tend to suppress neuronal responses of stimuli within the RF center with similar features. SS plays a crucial role in visual perception, for example in segmentation of object boundaries, visual saliency, and rapid figure-ground segmentation of moving objects (Petrov and McKee, 2006; Nurminen and Angelucci, 2014; Bijanzadeh et al., 2018; Tadin et al., 2019). Feedforward and feedback connections between V1 and higher brain regions, V1 intracortical horizontal connections, interlaminar connections, and disinhibitory circuits have been identified to account for the generation of SS (Angelucci et al., 2002, 2017; Angelucci and Bressloff, 2006; Shushruth et al., 2013; Bijanzadeh et al., 2018; Nurminen et al., 2018; Keller et al., 2020).

Interestingly, the SS change has been linked to an alteration in GABA-ergic inhibitory cortical function (Angelucci and Bressloff, 2006; Angelucci et al., 2017). Much weaker surround suppression effects were found in human subjects with decline in efficacy of cortical GABA-ergic inhibitory systems, such as patients with schizophrenia and major depression (Tadin et al., 2006; Golomb et al., 2009), while magnetic resonance spectroscopy has provided additional evidence (Yoon et al., 2010).

**FIGURE 1:**
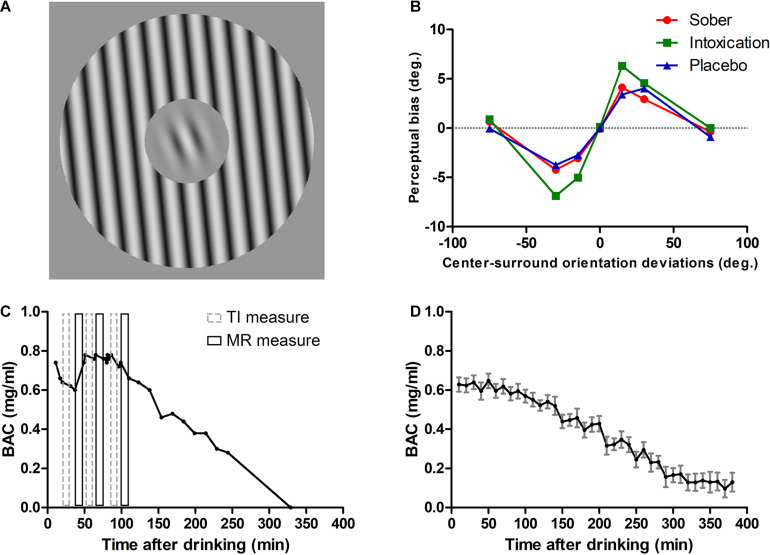
Stimuli, TI results for one participant and profiles of alcohol metabolism. **(A)** TI measure was an orientation discrimination task. Stimuli have a center-surround configuration; the center Gabor target is surrounded by a grating annulus which could have an orientation of 0°, ± 15°, ± 30°, ± 75° with respect to the center Gabor (in the illustration it is +15°). Participants were required to report whether the Gabor orientation was CW or CCW from the internal vertical. **(B)** Results of a participant. Tilt repulsion effects under sober (red solid line with filled circle), alcohol intoxication (green solid line with filled rectangle), and placebo (blue solid line with filled triangle) states. **(C)** BAC as a function of time after drinking for the same participant. Gray dashed rectangles represent the three TI measurements and black solid rectangles represent the three MR measurements. **(D)** Mean BAC curve across all participants (computed by pooling BAC values within 10 min bins). Error bars represent SEM.

Tilt illusion (TI) is a type of SS effect, where the perceived orientation of the center target is biased by the simultaneously presented surround stimulus (Figure 1A). In particular, subjects strongly misperceive the physical orientation of the center target when the surround orientation has an angular difference between 0° and 50° (repulsion effect), while a systematic weaker effect is observed for differences around 75° (attraction effect) (example in Figure 1B; O’Toole and Wenderoth, 1977; Wenderoth and Smith, 1999; Takao et al., 2020). Several researchers have proposed that repulsive TI is caused by lateral inhibition from spatially arranged orientation hypercolumns of V1 neurons (Blakemor et al., 1970, 1973; Kapadia et al., 2000; Qiu et al., 2013; Takao et al., 2020) and successfully modeled with such assumptions (Gilbert and Wiesel, 1990; Bednar and Miikkulainen, 2000; Tzvetanov, 2012). On the other hand, the attractive TI is attributed to more global orientation mechanisms, related to higher-level extra-striatal orientation processing (Wenderoth and Johnstone, 1988; Smith and Wenderoth, 1999). Dynamic causal modeling of fMRI signals further suggested that perception of the repulsive TI reflects an intra-hemispheric integration mechanism in V1 (Song and Rees, 2018). Therefore, repulsive TI measures are considered, from psychophysics, physiological, and modeling perspectives to reflect the inhibitory response properties of V1.

Given that ethanol could potentiate GABA_*A*_ receptors and promotes the spontaneous release of GABA (Abrahao et al., 2017), this should increase overall inhibition. Therefore, we hypothesized that moderate alcohol consumption would alter visual perception related to V1 by increasing perceptual effects attributed to inhibition. We investigated this hypothesis by using the center-surround TI effect as a probe of inhibition changes, such that the potentiation of the inhibitory system should increase the magnitude of TI. Participants in our current research performed TI measurements under sober, alcohol intoxication [approximately 0.6 mg/ml of blood alcohol concentration (BAC)], and placebo conditions, with a within-subjects and double-blind experiment design.

## Materials and Methods

### Brief Experimental Design

The experimental design has been described in detail in our previous publication (Wang et al., 2018, 2019). In brief, a total of 33 university students and staff (24 males, 20–30 years old, mean = 23.94 years) participated in our research. This research was approved by the Ethics Committee of the University of Science and Technology of China and followed the guidelines of the Declaration of Helsinki. Written informed consent was obtained from each participant after explanation of the nature and possible consequences of the study.

A within-subjects and double-blind design was used in the current research. Each subject was exposed to three experimental conditions (sober, placebo and moderate alcohol) on separate days. The dose of moderate alcohol was approximately 0.6 mg/ml, and the amount of liquor was calculated based on individual participant’s age, gender, body height and weight (Watson et al., 1980; Stock et al., 2017). The sober measure was always conducted first, the order of placebo and alcohol was counterbalanced. During each experimental condition, participants performed, in an interleaved manner, a visual direction (MR) and orientation (TI) discrimination task, and these discrimination tasks were conducted three times in the alcohol condition (Figure 1C). In total, each participant performed up to five MR measurements and five TI measurements, for those who could complete the whole experiment. In the present work only the TI data are presented.

### Stimulus and Procedure

A daily calibrated CRT monitor (Sony G520; 85 Hz, resolution of 1,600 × 1,200 pixels) was used to display the stimulus which was generated by custom written Matlab functions (Mathworks Inc.) using the Psychophysics toolbox (Brainard, 1997; Pelli, 1997). The eye-to-screen distance was maintained with a chin rest and fixed at 1.5 m. Luminance values were obtained from a 256 RGB gray levels look-up table.

The stimulus used in TI measurements was a center-surround configuration grating with seven different surround orientations (0°, ± 15°, ± 30°, ± 75°; angle was defined with respect to the orientation of the center test; Figure 1A). The orientation of the central Gabor grating varied around the vertical from trial to trial to measure participants’ perceived verticality (defined as 0°). The diameter of center grating was 1.33° and the width of surround annulus was equal to the center diameter. The stimulus had a spatial frequency of 3 cycle/°, both center and surround had strong contrast (90%) in order to measure inhibition related TI (Tzvetanov, 2012), and was presented on a mean background luminance of 35 cd/m^2^. The center orientation varied according to the weighted up-down rule (Kaernbach, 1991) with steps up/down of 5/2 and 2/5 degrees with base step of 1°. Staircases started with orientation of −21°/+21° at the opposite side of the convergence point, allowing rapid measures within the transition region of the psychometric function. Each staircase had 40 trials.

There were 560 trials (80 trials × 7 surround directions) in each TI measurement. In each trial, the stimulus was presented for 200 ms after a 200 ms fixation. Participants had to report whether the center target grating orientation tilted clockwise (CW) or counterclockwise (CCW) from the internal vertical orientation by pressing corresponding keys on the computer keyboard. No feedback was provided regarding response correctness.

### Alcohol Administration and BAC Measurement

Subjects consumed an individual amount of liquor (40% volume ethanol) mixed with equal proportions of orange juice to reach a BAC, when assuming an absorption deficit. The amount of liquor for each subject was calculated based on previous research (Watson et al., 1980; Stock et al., 2017):

(1) $$\text{c}=\frac{0.8\times A}{1.055\times T\,B\,W}$$

where c is the maximum possible BAC milliliter and was set to 1.5. Since this equation does not take the absorption deficit into account, the final BAC value for each subject was determined by an Alcotest measurement. *A* is the amount of alcohol in grams that must be consumed. *TBW* is the total body water in liters and was estimated using different equations for men and women to account for differences in body fat:

(2) $$T\,B\,W_{w\,o\,m\,e\,n}=-2.097+\left(0.1069\times h\right)+\left(0.2466\times w\right)$$

(3) $$T\,B\,W_{m\,e\,n}=2.447-\left(0.09516\times a\right)+\left(0.1069\times h\right)+\left(0.2466\times w\right)$$

where *h* is the body height in cm, *w*is the body weight in kg, and *a* is the age in years.

Finally, the amount of alcoholic beverage in ml (V) was calculated using the following equation:

(4) $$\text{V}=\frac{A}{\left(v\,o\,l÷100\right)\times 0.8}$$

where *vol* is the % volume of the alcoholic beverage and was set to 40. V is the final amount (ml) of alcohol that subjects consumed in the experiment. The same volume of juice was mixed with alcohol for administration. Irrespective of the individual amount, subjects were asked to ingest the liquor within 15 min.

Before the experiment began, the BAC was measured with an Alcotest 6510 breathalyzer (Drägerwerk, Lübeck, Germany) to ensure a BAC of 0 mg/ml. The BAC was measured before and after each block of measurement, starting 10 min after consumption of all alcoholic beverages. After all tasks were done or stopped, additional BAC measures were carried approximately every 10–30 min until the level faded to zero and thus allowed the subjects to recover from alcohol effects. The average BAC levels near the three TI measures were 0.60 ± 0.15 mg/ml (mean ± SD), 0.63 ± 0.18 mg/ml, 0.60 ± 0.13 mg/ml, respectively. (see Figures 1C,D for an individual and across subjects mean BAC curves, respectively).

### Psychometric Function Fitting

We used the same method described in previous MR research (Wang et al., 2018) to analyze the TI data. Briefly, for each surround orientation, we fitted the probability of clockwise responses to center orientation θ with the following psychometric function:

(5) $$\text{p}\,\left(\theta \right)=l+\frac{1-2\,l}{1+e\,x\,p\,\left(-\frac{l\,o\,g\,\left(21/4\right)}{\sigma }\,\left(\theta -\mu \right)\right)}$$

where *l* is the participant’s lapse rate, and μ and σ are the perceived vertical orientation (also called “bias”) for the given surround and the threshold of the subject for perceiving a deviation from verticality (>84% correct responses), respectively. The function was adjusted to TI data by using Bayesian fitting (Treutwein and Strasburger, 1999). Prior parameters were: *l*-beta probability distribution with parameters Beta (1.2, 15); σ-gamma probability distribution with parameters Gamma (2.5, 2.5); and μ had a uniform prior. The bias values of a given block of measures were then adjusted to a mean of zero by subtracting their average. Log10 values of lapse rate were used in statistical analysis.

### Statistical Analysis

Repeated measures ANOVA was conducted to compare bias, threshold, and lapse rate, with the test surround orientations (4 levels: 0°, ± 15°, ± 30°, ± 75°) and different conditions (3 levels: sober, placebo, and alcohol) as the within-subject factor as well as with the Geisser-Greenhouse adjusted statistics (epsilon is reported as ehat). Bias used in this statistical test was the half-difference between two opposite surround orientations, while threshold and lapse rate were mean values of the two symmetric surround orientations. We also performed Bonferroni *post-hoc* multiple comparisons for the repulsion strengths at each test orientation.

### Data Analysis

From the 33 participants, 5 participants did not have a full data set for TI measurements (at least one alcohol measure, or sober, or placebo measures) for one of the following reasons: did not want to drink such amount of alcohol and the decision was taken to abort measures with these persons (3 subjects), missed Control measures due to availability of the persons (1 subject), and left to another city for work (1 subject). The number of alcohol measures depended on the individual subject’s well-being during those measures. After checking the Bayesian fit results, one person had 4 out of 7 high lapse rates (above 0.20) in the first alcohol measurements. Inspection of the corresponding staircases of these measurements showed that this participant responded somewhat randomly starting from around the middle of the measurement block. Therefore, we also excluded the data of this participant from the analysis. Among the remaining participants, 27 participants had at least one alcohol measure, 26 participants had at least two alcohol measures, 20 participants had three alcohol measures.

## Results

In the following, we emphasize the results of the second TI measures because they were obtained around the peak intoxication level (see Wang et al., 2018), where we expected the strongest Intoxication effect (Figures 1C,D). Then, we briefly present the results of the first and third measurements, which provide qualitatively similar conclusions.

### Increased Repulsive TI Effect After Alcohol Administration

**FIGURE 2:**
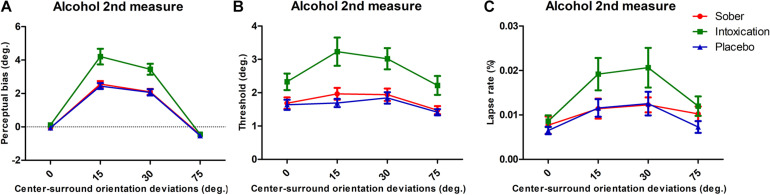
Tilt repulsion results and lapse rates of sober (red), alcohol intoxication (green), and placebo (blue) conditions. **(A)** TI effects, indicated by the perceptual bias necessary to perceive the center as vertical, as a function of center-surround orientation deviations (positive values indicate tilt repulsion of the surround; the results for CW and CCW surrounds of same angular deviation were pooled). **(B)** Orientation thresholds around perceived verticality. The mean values for the vertical discrimination thresholds as a function of the experimental condition. **(C)** Lapse rates of various surround orientations. Error bars represent SEM.

TI effect was measured under each condition for all participants. The perceived orientation was misjudged as expected from previous reports. In all three conditions, the TI effect had similar patterns with the surround grating orientation systematically modulating the amount of center orientation misperception. The misperception was more pronounced when the angular difference between the center and surround grating were 15° and 30°, and we observed a small attraction effect at 75°. The alcohol intoxication markedly increased the TI magnitude (Figure 2A). We performed repeated measures of ANOVA with Surround Orientation factor (0°, ± 15°, ± 30°, ± 75°) and Condition factor (sober, placebo, and alcohol). Results showed that there were significant main effects of center-surround orientation differences on tilt illusion [*F*(3, 75) = 172.55, *p* < 0.001, ehat = 0.63] and the condition [*F*(2, 50) = 15.21, *p* = 0.0004, ehat = 0.55], as well as a significant interaction between them [*F*(6, 150) = 12.45, *p* = 0.0001, ehat = 0.29]. This interaction effect was driven by a significant TI increase due to alcohol intoxication at surround orientation of 15° and 30°. We then conducted a paired *t*-test to identify differences at each surround orientation, under the placebo and intoxication conditions. Compared to the placebo condition, the intoxication condition had significantly higher amplitudes at a surround orientation of 15° [*t*(25) = 3.80, *p* < 0.001] and 30°[*t*(25) = 4.74, *p* < 0.001], but not at 0° [*t*(25) = 1.41, *p* = 0.1717] and 75° [*t*(25) = 0.71, *p* = 0.4838] (Bonferroni correction of significance level to 0.05/4 = 0.0125). Importantly, the attractive TI effect, despite its small value, was systematically present across all three conditions (*t*-test comparison to 0° reference, all *p* < 0.01).

### Worsened Discrimination Performance

We extracted discrimination thresholds from the psychometric functions in order to characterize the influence of alcohol intoxication on participants’ orientation discrimination. These thresholds described the deviation of the orientation from the perceived vertical at which participants reported reliable deviations in 84% of trials. These deviations reflect the difficulty of discriminating two close orientations of grating, with higher values indicating a worsened discrimination ability. The average thresholds for each experimental condition are presented in Figure 2B. They were modulated by Condition [*F*(2, 50) = 15.80, *p* = 0.0002, ehat = 0.59] and Surround Orientation [*F*(3, 75) = 16.42, *p* < 0.001, ehat = 0.93]. There was a significant interaction between the two factors [*F*(6, 150) = 2.65, *p* = 0.0413, ehat = 0.63].

### High Level Effects

We obtained lapse rates from the psychometric function fits. They represent asymptotic performances of the participants for the strongest stimuli, at which values the task is easy to perform and therefore allows to measure subjects’ full attentional deployment. Variation of the lapse rate between conditions is thus interpreted as strong evidence of attentional load changes (Ling and Carrasco, 2006).

The average lapse rates for each experimental condition are presented in Figure 2C. We observed effects on lapse rates under conditions and surround orientations [conditions, *F*(2, 50) = 7.51, *p* = 0.0028, ehat = 0.83; surround orientations, *F*(3, 75) = 14.43, *p* < 0.001, ehat = 0.94], but no interaction effect between them [*F*(6, 150) = 0.75, *p* = 0.5946, ehat = 0.86]. We interpret these results as indicating that alcohol intoxication limited participants’ overall attentional state (for example decreasing their transient attention level; Ling and Carrasco, 2006).

### Simultaneous Effects on Bias and Threshold

We further checked that effects were not visible through possible simultaneous changes of repulsive TI effects and discrimination thresholds (Solomon and Morgan, 2009) specifically affecting the Alcohol condition. Pearson’s correlations were conducted between perceived biases and thresholds (12 correlations, 3 conditions × 4 surrounds). Only four correlations passed the statistic tests (sober at 30°: *r* = 0.66, *p* < 0.0001; alcohol at 15°: *r* = 0.82, *p* < 0.0001 and at 30°: *r* = 0.78, *p* < 0.0001; placebo at 30°: *r* = 0.65, *p* < 0.0001; Bonferroni adjustment to 0.05/12 = 0.0042; all other comparisons with *p* > 0.01) (see Supplementary Figure 1). Thus, we consider that the intoxicated condition did not simultaneously influence in a particular manner perceived bias and threshold due to either low-sensory effects or high-cognitive effects.

### Results of the First and Third Alcohol Measurements

**FIGURE 3:**
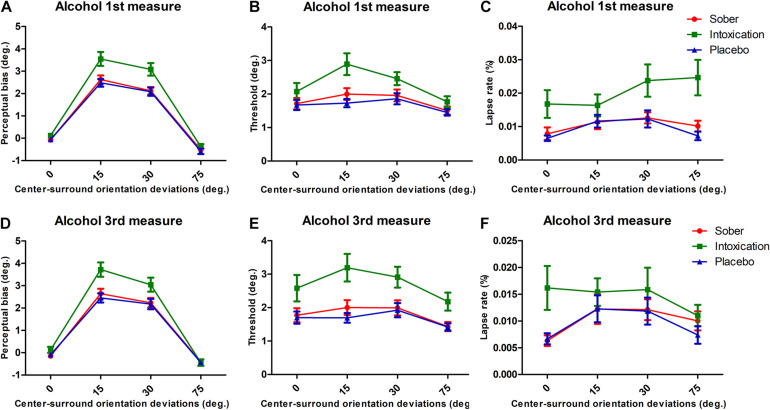
Results for bias, threshold, and lapse rates for 1st and 3rd measurements of alcohol. Same format as Figure 2.

Data from the first and third alcohol measures were analyzed in the same manner. They showed consistent effects of alcohol intoxication as with the second alcohol measurement. Figures 3A-F presents the results. The corresponding ANOVA results are presented in Table 1. We note that (1) the Bias measures Condition always showed significant effects with the Alcohol measures giving stronger repulsion, and an interaction between Condition with Surround Orientation, (2) Thresholds were always higher in the Alcohol measure, (3) lapse rates were always higher in alcohol condition, and there were no interactions between Condition and Surround Orientations.

**TABLE 1:** ANOVA table results for each alcohol measure.

	Measure	Condition	df	F	Sig.	Ehat
First alcohol measure (*n* = 27)	Bias	C	2,52	25.63	< 0.001	0.650
		S	3,78	158.47	< 0.001	0.521
		C*S	6,156	6.93	0.0003	0.513
	Threshold	C	2,52	17.47	<0.001	0.793
		S	3,78	15.81	<0.001	0.758
		C*S	6,156	2.89	0.0316	0.594
	Lapse	C	2,52	13.59	0.0002	0.721
		S	3,78	9.42	< 0.001	0.956
		C*S	6,156	1.36	0.2446	0.815
Second alcohol measure (*n* = 26)	Bias	C	2,50	15.21	0.0004	0.545
		S	3,75	172.55	<0.001	0.628
		C*S	6,150	12.45	0.0001	0.294
	Threshold	C	2,50	15.80	0.0002	0.587
		S	3,75	16.42	<0.001	0.927
		C*S	6,150	2.65	0.0413	0.627
	Lapse	C	2,50	7.51	0.0028	0.829
		S	3,75	14.43	<0.001	0.935
		C*S	6,150	0.75	0.5946	0.859
Third alcohol measure (*n* = 20)	Bias	C	2,38	11.39	0.0014	0.625
		S	3,57	146.24	<0.001	0.594
		C*S	6,114	7.78	0.0001	0.546
	Threshold	C	2,38	14.19	0.0004	0.636
		S	3,57	9.58	0.0001	0.804
		C*S	6,114	1.52	0.2135	0.561
	Lapse	C	2,38	6.06	0.0077	0.869
		S	3,57	6.46	0.0018	0.826
		C*S	6,114	1.27	0.2877	0.681

*^∗^It means the interaction effects between surround orientation (S) and condition (C).*

## Discussion

In this study, we investigated the putative effects of alcohol onto V1 inhibitory activity by using the center-surround repulsive TI effect as a probe of inhibitory contextual interactions. It was measured under acute moderate alcohol ingestion, and contrasted to sober and placebo conditions. We observed obvious increases in the amount of repulsive TI after alcohol ingestion together with the discrimination threshold of orientation, both effects strongly supporting increased inhibitory surround interactions.

For several decades lateral inhibition between orientation sensitive neurons in V1 was proposed to account for the repulsive TI (Blakemor et al., 1970, 1973; Georgeson, 1973; Gilbert and Wiesel, 1990; Takao et al., 2020). The psychophysics observations in the center-surround configuration are considered to reflect the particular organization of spatial lateral interactions in V1, where inhibition created by the presence of stimuli beyond the classical RF is very common (Shushruth et al., 2013; Angelucci et al., 2017). Because alcohol consumption increases inhibition strength in area V1 (Chen et al., 2010), we propose that it also increases lateral inhibition. This is consistent with our alcohol-related observations of stronger repulsive TI and increased overall orientation discrimination thresholds.

Previous research has investigated plausible lateral inhibition changes under alcohol intoxication by using psychophysical measurements of “Westheimer” functions (a probe of retinal center-surround interactions) or the Hermann grid illusion (Johnston and Timney, 2008, 2013). Although a full analysis with the signal detection theory of the Westheimer function under alcohol effects is still missing (Timney et al., 2016), the “Westheimer” function results (Johnston and Timney, 2008) seem to comfort our findings of increased lateral inhibition. For the Hermann grid illusion, where people see illusory black dots on the crossings of vertical and horizontal thin white stripes drawn over a black/gray background (or vice versa), its interpretation as a probe of retinal or subcortical lateral inhibition was clearly dismissed (Schiller and Carvey, 2005; Geier et al., 2008) and thus the reported results must be carefully considered. In contrast, our within-subject simple orientation discrimination task design, complete psychometric function measure, and well characterized center-surround inhibitory effect of TI provide sufficient and reliable evidence to prove that lateral inhibition is enhanced by alcohol in area V1.

One important aspect of our data is that it allows to discard specific explanations of alcohol induced repulsive TI due to higher-level effects of orientation processing or attentional changes targeting the exact conditions where the repulsive TI appears. TI patterns were systematically modulated by surround orientations consistently across all conditions, that means participants reliably represented individual perceptual sensitivities even under alcohol intoxication. The increase in perceptual bias only occurred at surround orientations of 15° and 30°, while the attractive TI effect was unchanged. The increased lapse rates across surround orientations indicated that subjects had global changes in attention to the task, and these “high cognitive” effects were unrelated to specific surround conditions. These results showed that deficits such as more global, higher-order, orientation processing is not visibly affected and deteriorated cognition, i.e., attention, represent generalized effects.

Another source of interference with our observations is that subjects might have an unstable fixation state in the alcohol condition, but that seems unlikely. Microsaccades are one of the main type of eye movements during visual fixation in humans (Martinez-Conde et al., 2004). Alcohol significantly increases saccade latency both in low (0.4 g/kg) and high (0.8 g/kg) dose in humans (Roche and King, 2010). After ethanol administration, monkeys showed diminution of the frequency of saccades and prolongation of fixation periods (Fuster et al., 1985). The acute alcohol ingestion increased the number and duration of fixations (mean and total) in humans during visual Maze test (Silva et al., 2017) and reading (Watten and Lie, 1997), and the mean eye fixation time was above 250 ms, longer than the stimulus presentation time (200 ms) in our experiment. From these reports, we concluded that eye movements are an unlikely source of specific TI increase in the alcohol condition.

One interesting feature in the TI effect is the strong correlation of the amount of TI bias and threshold of discrimination (Solomon and Morgan, 2006, 2009) at oblique surround angles. This phenomenon in the data led Solomon and Morgan (2006) to propose that both changes in perceived value and acuity (inverse of threshold) can be explained by a stochastic recalibration mechanism, which is indistinguishable from plausible increased internal noise (Solomon and Morgan, 2006). While this explanation is debatable due to the decrease of internal background noise by alcohol administration (Chen et al., 2010), we would like to emphasize that increased inhibition also allows to explain simultaneous changes in both variables, of perceived value and threshold (Tzvetanov and Womelsdorf, 2008, their Figure 5, for an application to motion direction). While the current analyses and experimental design may seem insufficient for disentangling between both possibilities, neurophysiological results (Chen et al., 2010) and literature reports (see section “Introduction”) hint to stronger inhibition.

Low-level based neuronal explanations of the TI effect showed that various population changes of center orientation tuning characteristics can contribute to the final TI effect: amplitude inhibition, tuning width change, shift of neuronal preferred orientation, etc. (Gilbert and Wiesel, 1990; Schwartz et al., 2007). In addition, orientation surround suppression measures, obtained with the probe of apparent contrast (Cannon and Fullenkamp, 1991) or more recently comparing human psychophysics and neurophysiology (Shushruth et al., 2013), hinted toward two distinct spatial mechanisms: one narrowly tuned that is spatially restricted and the other broadly tuned that is spatially widespread. From the reported alcohol effects onto neuronal tuning properties (Chen et al., 2010), we consider, for the moment, that the alcohol-enhanced repulsive TI effect comes from a stronger surround amplitude of inhibition due to alcohol, until further evidences are available.

From a larger perspective, the GABA-ergic inhibitory system plays critical roles in V1 functions and their putatively associated visual perceptions: GABA agonists improve V1 function in senescent monkeys (Leventhal et al., 2003); it increases responsiveness and controls response gain in V1 (Katzner et al., 2011); its modulation affects spatio-temporal contrast sensitivity of healthy subjects (Blin et al., 1993) and binocular rivalry in autism patients (Spiegel et al., 2019) that have reduced GABA-ergic action (Robertson et al., 2016). Among various inhibitory neuronal subclasses, activation of parvalbumin-expressing (PV) interneuron improves neuronal feature selectivity, perceptual discrimination and response reliability in V1 (Lee et al., 2012; Zhu et al., 2015), somatostain-expressing (SOM) interneuron in V1 sharpen neuronal feature selectivity and has contrast tuning (Wilson et al., 2012; Millman et al., 2020), and vasoactive intestinal peptide-expressing (VIP) interneuron enhances responses to weak but specific stimuli in V1 (Millman et al., 2020). These interneuronal subclasses also contribute to SS (Angelucci et al., 2017). For example, the disinhibitory circuit that consists of SOM and VIP neurons regulates contextual modulation in V1 (Keller et al., 2020). Recently, a research has linked the SS to the inhibitory-based neural responses in human V1, proved by enhanced Gamma oscillations which can reflect the activation of inhibitory neurons (Orekhova et al., 2020). In our study, we hypothesized to increase inhibition strength in human brain by moderate alcohol intoxication and thus to observe enhanced SS via the repulsive TI probe. Our results provide strong psychophysics evidence linking GABA-ergic inhibition in V1 to visual perception, consistent with the above research.

In summary, we think that our data altogether demonstrates that alcohol consumption induced stronger repulsive TI that reflects a specific change at very early neural stages of orientation processing, as V1. In line of the findings about enhancement of GABA_*A*_ receptors and spontaneous release of GABA by ethanol (Abrahao et al., 2017), we propose that the V1 GABA-ergic system could account for the observed visual perception changes reported in our study. These results combined with our previous report in motion domain (Wang et al., 2018) show that alcohol enhances surround suppression effects at various hierarchical stages of the visual system, which could be explained by increased inhibitory processing in motion- or orientation-sensitive areas.

## Data Availability Statement

The raw data supporting the conclusions of this article will be made available by the authors, without undue reservation.

## Ethics Statement

The studies involving human participants were reviewed and approved by the Ethics Committee of the University of Science and Technology of China. The patients/participants provided their written informed consent to participate in this study.

## Author Contributions

ZW and HW designed behavioral experiments and performed experiments. TT and HW performed data analysis and wrote the manuscript. ZW, YZ, and TT provided project supervision and funds. All authors discussed and commented on the manuscript and critically reviewed content and approved the final version for publication.

## Conflict of Interest

TT is currently working for his own company NEUROPSYPHY Tzvetomir TZVETANOV EIRL. The remaining authors declare that the research was conducted in the absence of any commercial or financial relationships that could be construed as a potential conflict of interest.
