# Supplementary material for: Moderate Alcohol Intake Changes Visual Perception by Enhancing V1 Inhibitory Surround Interactions
Source: Front Neurosci. 2021 Jul 5;15:682229. doi: 10.3389/fnins.2021.682229 (PMC8287857; doi:10.3389/fnins.2021.682229)
Supplement: Supplementary file 1 [file Table_1.DOCX]

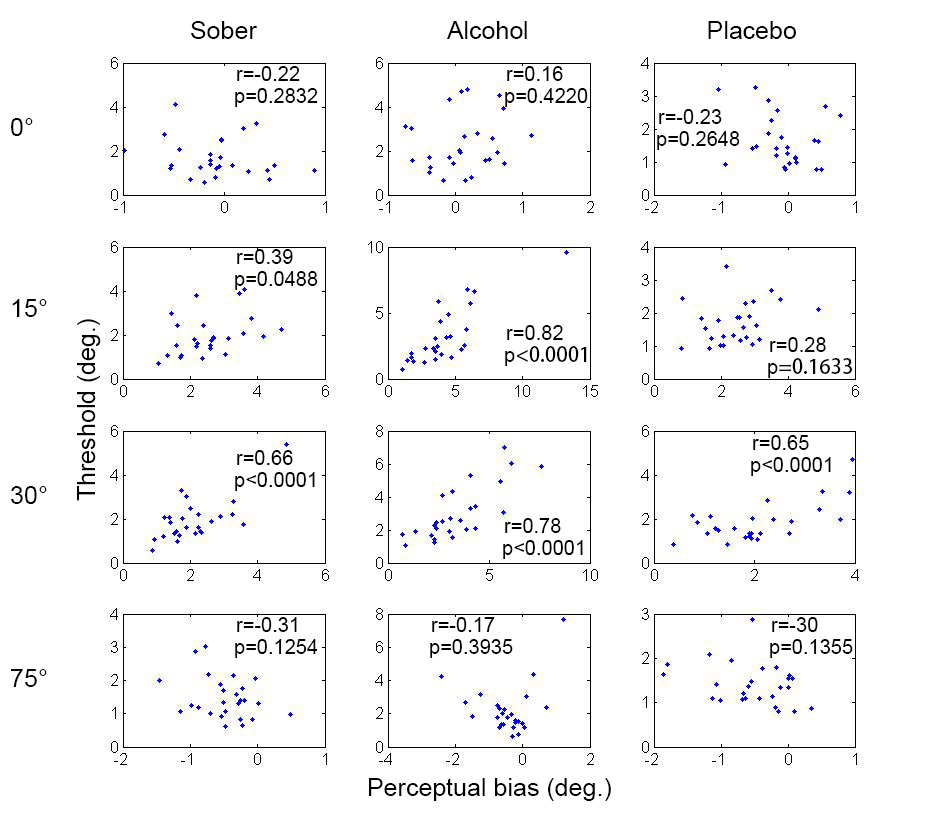


**Supplementary Figure 1.** Pearson correlation plots between perceptual bias and threshold for each surround orientation under each condition. Each blue point stands for one subject. The ordinate is the threshold, and the abscissa is the perceptual bias.
